# Multi-study *R*-learner for estimating heterogeneous treatment effects across studies using statistical machine learning

**DOI:** 10.1093/biostatistics/kxaf040

**Published:** 2025-12-18

**Authors:** Cathy Shyr, Boyu Ren, Prasad Patil, Giovanni Parmigiani

**Affiliations:** Department of Biomedical Informatics, Vanderbilt University Medical Center, 2525 West End Avenue, Nashville, TN 37203, United States; Laboratory for Psychiatric Biostatistics, McLean Hospital, 115 Mills Street, Belmont, MA 02478, United States; Department of Biostatistics, Boston University School of Public Health, 715 Albany Street, Boston, MA 02115, United States; Department of Data Science, Dana-Farber Cancer Institute, 450 Brookline Avenue, Boston, MA 02215, United States

**Keywords:** causal inference, conditional average treatment effect, machine learning, between-study heterogeneity, precision medicine

## Abstract

Heterogeneous treatment effect (HTE) refers to the nonrandom, explainable variation in treatment effects for individuals in a population. HTE estimation is central to precision medicine, where accurate effect estimates can inform personalized treatment decisions. In practice, patients can present with covariate profiles that overlap with multiple studies, raising the challenge of optimally informing treatment decisions in a multi-study setting. We proposed a flexible statistical machine learning (ML) framework, the multi-study $ R $-learner, that leverages multiple studies to estimate the HTE. Existing multi-study approaches often assume that study-specific (i) conditional average treatment effect (CATE), (ii) expected potential outcome under no treatment given covariates, and (iii) treatment assignment mechanism are identical across studies, but these assumptions may not hold in practice due to differences in study populations, protocols, or designs. To this end, we developed our framework to directly account for these three types of between-study heterogeneity. It builds upon recent advances in cross-study learning and uses a data-adaptive objective function to combine cross-study estimates of nuisance functions with study-specific CATEs via membership probabilities, which enable information to be borrowed across studies. The multi-study $ R $-learner extends the $ R $-learner to the multi-study setting and is flexible in its ability to incorporate ML techniques. In the series estimation framework, we showed that the proposed method is asymptotically normal and more efficient than the $ R $-learner when there is between-study heterogeneity in the treatment assignment mechanisms. We illustrated using cancer data from randomized controlled trials and observational studies that the multi-study $ R $-learner performs favorably in the presence of between-study heterogeneity.

## 1. INTRODUCTION

Heterogeneous treatment effect (HTE) occurs when treatment effects vary across patients due to differences in patient characteristics, such as genetic or social factors. HTE estimation is key in precision medicine, where tailoring treatments to patients relies on accurate estimates to inform personalized treatment decisions. These decisions are important to many stakeholders, including patients and policymakers, for improving outcomes and guiding evidence-based health policies.

Recent facilitation of systematic data sharing initiatives provides opportunities for HTE estimation across multiple studies (Colnet et al. 2022). For example, curated datasets like curatedBreastData compiled high-quality studies, including both randomized controlled trials (RCTs) and observational studies, that measured the same treatment, outcome, and covariates in cancer patients (Planey 2020). Though access to multiple studies is promising due to larger sample size, leveraging them for HTE estimation can be challenging in real-world settings. In practice, a new patient can present with a covariate profile that overlaps with multiple study cohorts, raising the challenge of choosing the appropriate source for learning the treatment effect that would be most relevant for informing treatment for this new patient. Therefore, our goal is to leverage data from multiple existing studies to inform the expected treatment effect given the new patient’s covariate profile. A key challenge, however, is accounting for between-study heterogeneity, as overlooking this can lead to sub-optimal treatment decisions.

In this paper, we focus on developing a method that addresses three sources of between-study heterogeneity commonly encountered in real-world settings: heterogeneity in the study-specific (i) conditional average treatment effect (CATE) functions, (ii) expected potential outcome under no treatment given covariates, and (iii) probability of treatment assignment given covariates.

1. *Between-study heterogeneity in the CATEs* can result from varying distributions of unobserved effect modifiers across studies (Degtiar and Rose 2023). For example, evidence shows that exercise level is an effect modifier for breast cancer treatment (Cannioto et al. 2021). In practice, exercise level may be unobserved across studies, either because it’s not routinely collected or due to challenges in data collection. Consequently, CATEs can differ between studies with varying distribution of unmeasured exercise levels. Existing methods commonly assume that study-specific CATEs are identical, or transportable, across studies (Stuart et al. 2001; Dahabreh et al. 2019; Cheng and Cai 2021), but this assumption may not always hold in practice.
2. *Between-study heterogeneity in expected potential outcome under no treatment conditional on covariates* can arise if the distributions of unmeasured covariates affecting the potential outcome under no treatment vary across studies. These unmeasured covariates influence the potential outcome under no treatment but not the treatment assignment, so they do not lead to unmeasured confounding. In our cancer example, different distributions of unmeasured exercise levels, which are also an effect modifier, can lead to between-study heterogeneity in breast cancer risk under no treatment (Hardefeldt et al. 2018). However, unmeasured exercise levels do not influence treatment assignment, so they’re not confounders.
3. *Between-study heterogeneity in the probability of treatment assignment given covariates* can arise due to differences in observed study-level factors (eg study design). For instance, in our breast cancer example, two independently sampled RCTs that measure the same covariates (eg biomarker, stage, etc.) can differ in their randomization ratios: one randomizes patients to arms in a 2:1 ratio, whereas the other uses a 1:1 ratio. Likewise, two observational cohorts can be drawn i.i.d. from distinct healthcare systems that differ in their observed treatment protocols, leading to different treatment propensity models. Dahabreh et al. (2020) proposed propensity score-based estimators that allow treatment assignment mechanisms to differ across RCTs. Dahabreh et al. (2019) and Tan et al. (2022) assumed that treatment assignment mechanisms are identical across studies. This assumption may not hold in observational studies, where treatment assignment often depends on patient characteristics and is rarely standardized across studies.

We propose a flexible multi-study machine learning (ML) framework for HTE estimation that directly accounts for between-study heterogeneity in the CATE, expected potential outcome under no treatment given covariates, and propensity score functions. Our framework, the multi-study $ R $-learner, extends Nie and Wager (2021)’s $ R $-learner to the multi-study setting. Briefly, the $ R $-learner estimates the HTE in a single study using a moment condition derived by Robinson (1988). This approach offers several advantages, including asymptotic guarantees, strong empirical performance, and algorithmic flexibility, making it ideal for extension to the multi-study setting. While traditional individual participant data meta-analysis can be used to estimate HTE across studies, it often relies on parametric models with limited flexibility. In contrast, the multi-study $ R $-learner can incorporate ML techniques for estimation, providing additional flexibility.

This paper is organized as follows: Section 2 formalizes the multi-study $ R $-learner framework. Section 3 establishes its asymptotic unbiasedness and normality, and effiency gain over the $ R $-learner in a two-study setting. Section 4 evaluates the method using simulated ovarian cancer data. Section 5 applies it to real-world breast cancer data from an RCT and observational studies.

## 2. METHODS

### 2.1. Problem setup

Suppose we have data on patients from $ K $ studies, indexed by $ k\,{=}\,1, \ldots, K $. For patient $ i\,{=}\,1, \ldots, n_{k} $ in study $ k $, we observe independent and identically distributed tuples $ (Y_{i},X_{i},A_{i},S_{i}=k) $ where $ Y $ denotes the outcome, $ X\in\mathcal{X}\subset\mathbb{R}^{p} $ the covariates, $ A\in\{0,1\} $ the treatment assignment, and $ S\in\{1, \ldots, K\} $ the random variable indicating study membership. We assume $ S $ is drawn according to $ p^{*}(k\mid x)=\mathrm{pr}(S\,{=}\,k\mid X\,{=}\,x) $, where $ k $ is the study label. We refer to $ p^{*}(k\mid x) $ as the membership probability of belonging to study $ k $ given covariate $ x $. Let $ n=\sum_{k\,{=}\,1}^{K}n_{k} $ denote the total sample size. Patients from different studies are assumed to be independent. We adopt the potential outcomes framework (Rubin 1974) and let $ \{Y(1),Y(0)\} $ denote the potential outcomes that would have been observed under each treatment. Our goal is to estimate the CATE function $ \tau^{*}(x)=E[Y(1)-Y(0)\mid X\,{=}\,x] $ using data from the $ K $ studies. Notably, $ \tau^{*}(x) $ is a weighted average of the study-specific CATE functions $ \tau^{*}_{k}(x)=E[Y(1)-Y(0)\mid X\,{=}\,x, S\,{=}\,k] $. To estimate $ \tau^{*}_{k}(\cdot), $ we make the following identifiability assumptions:

Assumption 1 (Consistency). $ Y\,{=}\,Y(1)A\,+\,Y(0)(1-A) $

Assumption 2 (Mean unconfoundedness within study). $ E[Y(a)\mid A\,{=}\,a, X\,{=}\,x, S\,{=}\,k]=E[Y(a)\mid X\,{=}\,x, S\,{=}\,k], $  *for every $ x $ with $ f(x, S\,{=}\,k) > 0, $ treatment $ a\in\{0,1\} $, and study $ k\,{=}\,1, \ldots, K $.*

Assumption 3 (Positivity of treatment assignment within study). *For each treatment $ a\in\{0,1\} $, pr$ (A\,{=}\,a\mid X\,{=}\,x, S\,{=}\,k) > 0 $ for each $ x $ with $ f(x, S\,{=}\,k) > 0 $ in study $ k\,{=}\,1, \ldots, K $.*

Assumption 1 states that $ Y $ is equal to the potential outcome under treatment actually received. Assumption 2 posits that there is no unmeasured confounding in the mean outcome function given $ x $ within each study. Assumption 3 holds by design in RCTs and will hold in observational studies if there is sufficient variability in the treatment within all possible values of $ x $.

### 2.2. Multi-study Robinson’s transformation

In the multi-study $ R $-learner, we extended Robinson’s transformation to the multi-study setting. Originally introduced by Robinson (1988) to estimate parametric components in partially linear models, this transformation has since gained renewed attention, with Nie and Wager (2021) using it to develop the $ R $-learner and Chernozhukov et al. (2018) highlighting it as a key example of double ML. We briefly summarize Robinson’s transformation in a single-study setting. We begin by letting $ m^{*}(x)=E[Y\mid X\,{=}\,x] $ denote the conditional mean outcome function. Note that $ m^{*}(x)=\mu^{(0)*}(x)+e^{*}(x)\tau^{*}(x), $ where $ \mu^{(0)*}(x)=E[Y(0)\mid X\,{=}\,x] $ denotes the expected potential outcome under no treatment conditional on covariates, and $ e^{*}(x)=\mathrm{pr}(A\,{=}\,1\mid X\,{=}\,x) $ the probability of treatment assignment given covariates (ie propensity score function). Under unconfoundedness, we have $ E[\epsilon\mid X, A]=0 $, where $ \epsilon=Y-\{\mu^{(0)*}(X)+W\tau^{*}(X)\} $ (Nie and Wager 2021). It follows that $ Y-m^{*}(X)=\{A-e^{*}(X)\}\tau^{*}(X)+\epsilon $, which is Robinson’s transformation. This transformation reformulates the estimation of $ \tau^{*} $ as a univariate residual-on-residual regression, where the dependent variable is the residual $ Y-m^{*}(X) $ and the predictor is the residual $ A-e^{*}(X) $. To extend it to a multi-study setting, we can re-write $ m^{*}(x) $ as $ \sum_{k\,{=}\,1}^{K}\{\mu_{k}^{(0)*}(x)+e^{*}_{k}(x)\tau^{*}_{k}(x)\}p^{*}(k\mid x), $ where $ \mu_{k}^{(0)*}(x)=E[Y(0)\mid X\,{=}\,x, S\,{=}\,k] $, and $ e^{*}_{k}(x)=\mathrm{pr}(A\,{=}\,1\mid X\,{=}\,x, S\,{=}\,k) $ are study-specific versions of $ \mu^{(0)*}(x) $ and $ e^{*}(x) $, respectively. Because $ m^{*}(\cdot) $ is a function of $ \tau^{*}_{k}(\cdot),\mu_{k}^{(0)*}(\cdot), $ and $ e^{*}_{k}(\cdot) $, it captures the between-study heterogeneity in the CATE functions, expected potential outcome under no treatment, and probability of treatment given covariates, respectively. Therefore, we want to account for this in our estimation. We let $ \epsilon=Y-\sum_{k\,{=}\,1}^{K}\left\{\mu_{k}^{(0)*}(X)+A\tau^{*}_{k}(X)\right\}p^{*}(k\mid X) $ denote the error term. Then, it follows that


(2.1) \begin{align*} Y-m^{*}(X)=\sum\limits_{k=1}^{K}\{A-e^{*}_{k}(X)\}\tau^{*}_{k}(X)p^{*}(k\mid X)+\epsilon, \end{align*}

which is the multi-study Robinson’s transformation.

Proposition 1. *Under Assumption 2, $ E[\epsilon\mid A, X]=0. $*

A proof is provided in the Supplementary Material. When there is no between-study heterogeneity in the CATEs, Equation (2.1) is equal to the single-study Robinson’s transformation. Thus, the $ R $-learner is a special case of the multi-study $ R $-learner, which we introduce in the next section.

### 2.3. Multi-study $ R $-learner and cross-study learning

The multi-study Robinson’s transformation in Equation (2.1) implies a duality between the estimation of the study-specific CATEs $ \tau^{*}_{1}(\cdot),\ldots, \tau^{*}_{K}(\cdot) $ and the regression problem with outcome $ Y-m^{*}(X) $ and covariate $ \{A-e^{*}_{k}(X)\}p^{*}(k\mid X) $ for $ k\,{=}\,1, \ldots, K $. Consider the multi-study oracle $ R $-loss, which is defined for known nuisance functions $ m^{*}(\cdot),e_{k}^{*}(\cdot) $ and $ p^{*}(k\mid\cdot) $ as


(2.2) \begin{align*} L_{n}\left(\tau_{1}(\cdot),\ldots, \tau_{K}(\cdot)\right)=\frac{1}{n}\sum\limits_{i=1} ^{n}\left[\{Y_{i}-m^{*}(X_{i})\}-\sum\limits_{k=1}^{K}\{A_{i}-e^{*}_{k}(X_{i})\}p^{*} (k\mid X_{i})\tau_{k}(X_{i})\right]^{2}.\end{align*}

Treating $ m^{*}(\cdot),e_{k}^{*}(\cdot) $ and $ p^{*}(k\mid\cdot) $ as nuisance functions enables estimation with flexible ML methods, as the Neyman orthogonality property of the $ R $-loss makes the moment condition locally insensitive to small errors in the nuisance estimates provided that they converge sufficiently fast. We formalize the required convergence assumption in Section 3.1. While these nuisance functions are generally unknown and need to be estimated from data, an exception is RCTs, where $ e^{*}_{k}(\cdot) $ is known by design (eg 0.5 in a 1:1 allocation trial). Hence, our approach can handle RCTs, observational studies, and a mix of both. To mitigate potential overfitting, we incorporate a regularizer $ \Lambda_{\tau_{k}} $ on the complexity of $ \tau_{k}(\cdot) $. Putting these pieces together, the optimal study-specific HTEs satisfy: $ \{\hat{\tau}_{1}(\cdot),\ldots, \hat{\tau}_{K}(\cdot)\}= $


(2.3) \begin{align*}\mathop{{\rm arg\, min}}_{{\tau}_{1},\ldots,{\tau}_{K}}\left\{\frac{1}{n}\sum\limits_{i=1}^{n}\left[\{Y_{i}-\hat{m}(X_{i})\}-\sum\limits_{k=1}^{K}\{A_{i}-\hat{e}_{k}(X_{i})\}\hat{p}(k\mid X_{i}){\tau}_{k}(X_{i})\right]^{2}+\Lambda_{\tau_{k}}\right\}.\end{align*}

In practice, multiple studies can have varying degrees of overlap in their covariate distributions. In Equation (2.3), the estimated membership probabilities $ \hat{p}(k\mid X) $ capture the degree of covariate overlap across studies $ k\,{=}\,1, \ldots, K. $ When there is no overlap between the studies’ covariate distributions, then no information is borrowed across studies through $ \hat{p}(k\mid X) $. An example is when a study’s exclusion criteria match the inclusion criteria of another exactly. Consider a pediatric vs. adult study where for $ X=\mathrm{age}, $  $ \hat{p}(\text{peds study}\mid age < 18)=1 $ and $ \hat{p}(\text{peds study}\mid age\geq 18)=0 $ for patients in the pediatric study and vice versa those in the adult study. On the other hand, when there is overlap in the covariate distributions, the estimated membership probabilities $ \hat{p}(k\mid X) $ enable information to be borrowed across studies through cross-study learning. To see this, suppose $ \tau^{*}_{k}(X)=X\beta_{k} $ for $ k\,{=}\,1,2 $. Let $ m^{*}_{k}(X)=E[Y\mid X, S\,{=}\,k] $ denote the mean outcome model in study $ k $. The contributions to the squared error term in Equation (2.3) are


\begin{align*}\begin{bmatrix}Y_{1}-\{\hat{m}_{1}(X_{1})\hat{p}(1\mid X_{1})+\hat{m}_{2}(X_{1})\hat{p}(2\mid X_{1})\}\\\vdots\\Y_{n_{1}}-\{\hat{m}_{1}(X_{n_{1}})\hat{p}(1\mid X_{n_{1}})+\hat{m}_{2}(X_{n_{1}})\hat{p}(2\mid X_{n_{1}})\}\\Y_{n_{1}+1}-\{\hat{m}_{1}(X_{n_{1}+1})\hat{p}(1\mid X_{n_{1}+1})+\hat{m}_{2}(X _{n_{1}+1})\hat{p}(2\mid X_{n_{1}+1})\}\\\vdots\\Y_{n}-\{\hat{m}_{1}(X_{n})\hat{p}(1\mid X_{n})+\hat{m}_{2}(X_{n})\hat{p}(2\mid X _{n})\}\end{bmatrix}\end{align*}

\begin{align*} -\begin{bmatrix}(A_{1}-\hat{e}_{1}(X_{1}))\hat{p}(1\mid X_{1})X_{1}&(A_{1}-\hat{e}_{2}(X_{1}))\hat{p}(2\mid X_{1})X_{1}\\\vdots\\(A_{n_{1}}-\hat{e}_{1}(X_{n_{1}}))\hat{p}(1\mid X_{n_{1}})X_{n_{1}}&(A_{n_{1}} -\hat{e}_{2}(X_{n_{1}}))\hat{p}(2\mid X_{n_{1}})X_{n_{1}}\\(A_{n_{1}+1}-\hat{e}_{1}(X_{n_{1}+1}))\hat{p}(1\mid X_{n_{1}+1})X_{n_{1}+1}&(A _{n_{1}+1}-\hat{e}_{2}(X_{n_{1}+1}))\hat{p}(2\mid X_{n_{1}+1})X_{n_{1}+1}\\\vdots\\(A_{n}-\hat{e}_{1}(X_{n}))\hat{p}(1\mid X_{n})X_{n}&(A_{n}-\hat{e}_{2}(X_{n}))\hat{p}(2\mid X_{n})X_{n}\end{bmatrix}\begin{bmatrix}\beta_{1}\\\beta_{2}\end{bmatrix},\end{align*}

Note that each patient’s contribution involves a weighted average of within-study and cross-study predictions. For example, for patients $ i\,{=}\,1, \ldots, n_{1} $ in study 1, $ \hat{m}_{1}(X_{i}) $ and $ \hat{e}_{1}(X_{i}) $ represent within-study predictions, and $ \hat{m}_{2}(X_{i}) $ and $ \hat{e}_{2}(X_{i}) $ cross-study predictions (and vice versa for patients in study 2). In other words, the multi-study $ R $-loss incorporates cross-study learning through the estimated membership probabilities $ \hat{p}(k\mid\cdot) $, linking cross-study estimates of nuisance functions ($ \hat{m}_{k}(\cdot) $ and $ \hat{e}_{k}(\cdot) $) with study-specific CATEs $ \tau_{k}(\cdot) $. The multi-study $ R $-loss can be thought of as shrinkage toward $ \tau(\cdot) $, and the membership probabilities control the degree of shrinkage. In summary, the multi-study $ R $-learner is a class of algorithms defined by three steps:

1. Estimate study-specific nuisance functions $ e^{*}_{k}(\cdot) $ and $ m^{*}_{k}(\cdot) $, $ k=1, \ldots, K $, by separate analyses of the $ K $ studies.
2. Estimate membership probabilities $ p^{*}(k\mid\cdot) $ by a pooled analysis of the $ K $ studies.
3. Estimate CATEs $ \tau^{*}_{k}(\cdot) $, $ k=1, \ldots, K $, using the multi-study version of Robinson’s transformation and a pooled analysis of the $ K $ studies.

To obtain $ \hat{m}_{k}(\cdot) $, $ \hat{e}_{k}(\cdot) $, and $ \hat{p}(k\mid\cdot) $, we use cross-fitting (van der Laan et al. 2011). We randomly divide $ n $ samples into $ Q $ evenly-sized folds, where $ Q $ is typically between 5 and 10. Let $ q(i)\in\{1, \ldots, Q\} $ denote the index of the fold that patient $ i $ belongs in. We denote the estimates of $ p^{*}(k\mid\cdot) $ based on all samples except for those in fold $ q(i) $ as $ \hat{p}^{-q(i)}(k\mid\cdot) $. To obtain cross-fitted estimates of study-specific quantities such as $ e^{*}_{k}(\cdot) $, the procedure is similar except we randomly divide the $ n_{k} $ samples from study $ k $ into $ Q $ evenly-sized folds. We let $ \hat{e}_{k}^{-q_{k}(i)}(\cdot) $ denote the estimate based on all $ n_{k} $ samples from study $ k $ except for those in $ q_{k}(i) $, where $ q_{k}(i)\in\{1, \ldots, Q\} $ denotes the index of the fold that patient $ i $ of study $ k $ belongs in. We minimize $ \hat{L}_{n}(\{\tau_{k}(\cdot)\}_{k\,{=}\,1}^{K})+\Lambda_{\tau_{k}} $ with respect to $ \{\tau_{k}(\cdot)\}_{k\,{=}\,1}^{K} $, where


(2.4) \begin{align*}\hat{L}_{n}\left(\{\tau_{k}(\cdot)\}_{k=1}^{K}\right)=\frac{1}{n}\sum\limits_{i=1}^{n}\left[\{Y_{i}-\hat{m}^{-q(i)}(X_{i})\}-\sum\limits_{k=1}^{K}\{A_{i}-\hat{e}^{-q_{k}(i)}_{k}(X_{i})\}\hat{p}^{-q(i)}(k\mid X_{i}){\tau}_{k}(X_{i})\right]^{2}\end{align*}

is the plug-in multi-study $ R $-loss. In the multi-study $ R $-learner framework, we estimate nuisance functions with cross-fitting in Steps 1 and 2 and plug the estimates into the multi-study $ R $-loss (2.4) to estimate study-specific CATEs in Step 3. To predict CATEs for new patients, given covariates $ x_{new}\in\mathcal{X} $, we compute $ \hat{\tau}(x_{new})=\sum_{k\,{=}\,1}^{K}\hat{\tau}_{k}(x_{new})\hat{p}(k\mid x_{new}) $.

## 3. THEORETICAL ANALYSIS

The goal of our theoretical analysis is 3-fold. First, we show that the difference between the oracle multi-study $ R $-loss $ L_{n}\left(\{\tau_{k}(\cdot)\}_{k\,{=}\,1}^{K}\right) $ in Equation (2.2) and the plug-in version $ \hat{L}_{n}\left(\{\tau_{k}(\cdot)\}_{k\,{=}\,1}^{K}\right) $ in Equation (2.4) diminishes at a relatively fast rate with $ n $. Second, we show that the multi-study $ R $-learner is asymptotically unbiased and normally distributed in the case of series estimation, a nonparametric regression method that approximates an unknown function using basis functions (Belloni et al. 2015). Series estimation is well-studied and serves as an ideal case for analyzing the asymptotic behavior of our method. Third, we show that the multi-study $ R $-learner is more efficient when there’s between-study heterogeneity in the propensity score functions.

Under the series estimation framework, the function $ f_{k}(x):=\tau^{*}_{k}(x) $ can be approximated by a linear combination of some pre-specified basis functions $ v_{k}(x)=(v_{k, 1}(x),\ldots, v_{k, d_{k}}(x))^{\intercal} $. That is, $ \tau^{*}_{k}(x)=v_{k}^{\intercal}(x)\beta_{k}+r_{f_{k}}, $ where $ \beta_{k}\in\mathbb{R}^{d_{k}} $ are the corresponding combination weights and $ r_{f_{k}} $ the approximation error for function $ f_{k} $. We allow $ d_{k} $ to grow with the sample size $ n_{k} $. The set of basis functions is not necessarily the same across studies; examples of these functions include polynomial and regression splines, among others. If the approximation error of $ \tau_{k}(x) $ is asymptotically negligible, then the oracle multi-study $ R $-loss in (2.2) can be expressed as a quadratic function of $ \beta=(\beta_{1}^{\intercal},\ldots, \beta_{K}^{\intercal})^{\intercal} $, $ L_{n}(\beta)=\frac{1}{n}\sum_{i\,{=}\,1}^{n}\left[\{Y_{i}-{m}^{*}(X_{i})\}-u_{i}^{\intercal}\beta\right]^{2}, $ where $ u_{i}=u(A_{i},X_{i})=W(A_{i},X_{i})Z(X_{i})v(X_{i}) $,


\begin{align*} W(A_{i},X_{i})=\mathrm{blkdiag}[\{W_{k}(A_{i},X_{i})\}_{k=1}^{K}], &\quad W_{k}(A_{i},X_{i})=\{A_{i}-e^{*}_{k}(X_{i})\}\mathbb{I}_{d_ {k}},\\Z(X_{i})=\mathrm{blkdiag}[\{Z_{k}(X_{i})\}_{k=1}^{K}], &\quad Z_{k}(X_{i})=p^{*}(k\mid X_{i})\mathbb{I}_{d_{k}},\end{align*}

$ v(x)=(v^{\intercal}_{1}(x),\ldots, v^{\intercal}_{K}(x))^{\intercal} $ , and $ \mathbb{I}_{n} $ is the $ n $-dimensional identity matrix. The plug-in multi-study $ R $-loss is $ \hat{L}_{n}(\beta)=\frac{1}{n}\sum_{i\,{=}\,1}^{n}\left[\{Y_{i}-\hat{m}^{-q(i)}(X_{i})\}-\hat{u}_{i}^{\intercal}\beta\right]^{2}, $ and estimates of $ \tau^{*}_{k}(\cdot) $, $ k\,{=}\,1, \ldots, K $, can be obtained by deriving the optimizer, $ \hat{\beta}={\rm arg\min}_{b}\frac{1}{n}\sum_{i\,{=}\,1}^{n}\left[\{Y_{i}-\hat{m}^{-q(i)}(X_{i})\}-\hat{u}_{i}^{\intercal}b\right]^{2}. $

### 3.1. Assumptions

To show that the multi-study $ R $-learner is asymptotically unbiased and normal, we make the following assumptions. We use $ a\lesssim b $ to denote $ a\leq cb $ for some constant $ c\,\gt\,0 $.

Assumption 4 (Boundedness). $ \left\|\tau^{*}_{k}(x)\right\|_{\infty} $ and $ E\left[\{Y-m^{*}(X)\}^{2}\mid X, A\right] $ are bounded, for any $ x\in\mathcal{X} $, $ A\in\{0,1\} $, $ k\,{=}\,1, \ldots, K $.

Assumption 5 (Estimation accuracy). $ E\left[\{m^{*}(X)-\hat{m}(X)\}^{2}\right] $, $ E\left[\{e^{*}_{k}(X)-\hat{e}_{k}(X)\}^{2}\right] $, and

$ E\left[\{p^{*}(k\mid X)-\hat{p}(k\mid X)\}^{2}\right] $ are $ O(a_{n}^{2}) $, where $ a_{n}=O(n^{-r}) $ with $ r\geq 1/4 $.

The assumptions above are required to show that the difference between the oracle and plug-in multi-study $ R $-loss diminishes at a relatively fast rate with $ n $. Assumption 4 is realistic when outcomes are inherently restricted to a finite range (eg in many real-world or clinical settings, effect sizes cannot grow indefinitely due to physical or biological constraints) or can be truncated/normalized to keep the variance bounded. Assumption 5 ensures that the nuisance functions are estimated with sufficient accuracy to guarantee valid asymptotic inference. ML methods that are known to achieve the required convergence rates under standard smoothness or sparsity conditions include penalized regression, random forests, boosting algorithms, and neural networks. To show asymptotic normality, we assume the membership probabilities follow a semi-parametric multinomial regression model, $ p^{*}(k\mid x)=\frac{exp(\tilde{v}_{k}(x)^{\intercal}\gamma_{k})}{1+\sum_{k\,{=}\,1} ^{K-1}exp(\tilde{v}_{k}(x)^{\intercal}\gamma_{k})} $ for $ k\,{=}\,1,2, \ldots, K-1 $, where $ \tilde{v}_{k}(x) $ are pre-specified basis functions for study $ k $, and $ \gamma_{k} $ is the corresponding parameter vector. We use this semi-parametric formulation to flexibly approximate the membership probabilities and protect against model misspecification by allowing complex, non-linear relationships between covariates and study membership through basis function construction. In practice, basis functions can be chosen based on domain knowledge (eg selecting clinically relevant covariates and transformations), and data-driven techniques (eg cross-validation, information criteria) can guide the selection and complexity of the basis functions. Let $ w_{i}=(y_{i},x_{i},a_{i},s_{i}) $ denote the vector of observations for the $ i $th patient. We are interested in the parameter vector $ \theta=(\beta, \gamma) $, where $ \gamma=\begin{bmatrix}\gamma_{1}^{\intercal},\gamma_{2}^{\intercal},\ldots, \gamma_{K-1}^{\intercal}\end{bmatrix}^{\intercal} $, that satisfies the system of equations, $ \Psi_{n}(\theta)=\frac{1}{n}\sum_{i\,{=}\,1}^{n}\psi(w_{i},\theta)=0, $ where $ \psi(w_{i},\theta)=\partial h(w_{i},\theta)/\partial\theta=\partial\left[\{y_{i}-m(x_{i})\}-u_{i}^{\intercal}\beta\right]^{2}/\partial\theta. $

Assumption 6 (Regularity). Let $ w $ be a vector taking values in $ \mathcal{W} $.

a) For each $ \theta\in\Theta $, $ h(\cdot, \theta) $ is a Borel measurable function on $ \mathcal{W} $.
b) For each $ w\in\mathcal{W} $, $ h(w, \cdot) $ is a continuous function over the compact parameter space $ \Theta $.
c) $ |h(w, \theta)|\leq b(w) $ for all $ \theta\in\Theta $, where $ b $ is a nonnegative function such that $ E[b(w)] \lt\infty. $

Assumption 7 (Identification). $ E[h(w, \theta_{0})] < E[h(w, \theta)] $ for all $ \theta\in\Theta $, $ \theta\neq\theta_{0}. $

Assumptions 6 and 7 allow us to establish consistency, ie $ \hat{\theta}\overset{p}{\rightarrow}\theta_{0}, $ where $ \hat{\theta}=\text{arg\, max}_{\theta\in\Theta}\frac{1}{n}\sum_{i\,{=}\,1}^{n}h(w_{i},\theta) $ and $ \theta_{0}=\text{arg\, max}_{\theta\in\Theta}E[h(w, \theta)]. $ Let $ H(w_{i},\theta)=\partial\psi(w_{i},\theta)/\partial\theta $ denote the Hessian. We now state the additional assumptions needed for establishing asymptotic normality.

Assumption 8 (Regularity).

a) $ \theta_{0} $ is an interior point of $ \Theta $.
b) $ \psi(w, \cdot) $ is continuously differentiable on the interior of $ \Theta $ for all $ w\in\mathcal{W} $.
c) Each element of $ H(w, \theta) $ is bounded in absolute value by $ b(w), $ where $ E[b(w)] \lt\infty $.
d) $ E[H(w, \theta_{0})] $ is positive definite.
e) $ E[\psi(w, \theta_{0})\psi^{\intercal}(w, \theta_{0})] \lt\infty $.

Assumption 9 (Approximation).

a) For each $ n_{k} $ and $ d_{k} $, there are finite constants $ c_{d_{k}} $ and $ l_{d_{k}} $ such that for each $ f_{k}\in\mathcal{G} $,
  \begin{align*}\left\|r_{f_{k}}\right\|_{F, 2}=\sqrt{\sum\limits_{a=0}^{1}\left\{\int_{x\in\mathcal{X}}r_{f_{k}}^{2}(a, x)dF(x)\right\}}\leq c_{d_{k}}\end{align*}
  and $ \left\|r_{f_{k}}\right\|_{F, \infty}=\sup_{x\in\mathcal{X},a\in\{0,1\}}\left|r_ {f_{k}}(a, x)\right|\leq l_{d_{k}}c_{d_{k}}, $ where $ \mathcal{G} $ is a function class.
b) $ \sup_{x\in\mathcal{X}}E[\epsilon^{2}I\{|\epsilon| > M\}\mid X\,{=}\,x]\rightarrow 0 $ as $ M\rightarrow\infty $ uniformly over $ n $.
c) Let $ \underline{\sigma}^{2}=\inf_{x\in\mathcal{X}}E[\epsilon^{2}\mid X\,{=}\,x] $. We assume that $ \underline{\sigma}^{2}\gtrsim 1 $.
d) Let $ \xi_{d_{k}}=\sup_{a, x}\left\|u(a, x)\right\| $, we assume that $ \{\xi_{d_{k}}^{2}\log(d_{k})/n_{k}\}^{1/2}\left(1+\sqrt{d_{k}}l_{d_{k}}c_{d_{k}}\right)\rightarrow 0, \\l_{d_{k}}c_{d_{k}}\rightarrow 0. $
e) $ \lim\limits_{n_{k}\to\infty}\sqrt{n_{k}/d_{k}}\cdot l_{d_{k}}c_{d_{k}}=0. $

Assumption 10 (Homoscedasticity). $ E[\epsilon^{2}\mid X, A, S\,{=}\,k]=\sigma^{2}_{k} $ and $ E[\epsilon^{2}\mid X, A]=\sigma^{2}. $

The constants $ c_{d_{k}} $ and $ l_{d_{k}} $ in Assumption 9a characterize the approximation properties of $ u(a, x)^{\intercal}\beta $. Assumption 9b is a mild uniform integrability condition, and it holds if for some $ m\,\gt\,2, \sup_{x\in\mathcal{X}}E[|\epsilon|^{m}\mid X\,{=}\,x]\lesssim 1 $. Assumptions 9d and 9e ensure that the impact of unknown design is negligible, and the approximation error is negligible relative to the estimation error, respectively. Specifically, the latter ensures that $ d_{k} $ grows with $ n_{k} $ at an appropriate rate such that the approximation error from the basis approximation is asymptotically negligible [see Belloni et al. (2015) for additional details on standard approximation theory]. Assumption 10 posits homoscedastic errors. Under Assumptions 1 and 2, the homoscedasticity assumption entails that $ \sigma^{2}=Var(Y\mid X, A)=\sum_{k\,{=}\,1}^{K}\sigma^{2}_{k}p(k\mid X)+Var(m_{S}(X)\mid X) $ does not vary with $ X $. For example, the first term will not depend on $ X $ if $ \sigma_{k} $ is identical across studies. The second term is homoscedastic for certain formulations of $ m_{S}(X), $ such as $ m_{S}(X)=f_{1}(S)+f_{2}(X) $ (ie random study-specific intercept model), where $ f_{1}(\cdot) $ and $ f_{2}(\cdot) $ denote functions.

### 3.2. Results

Lemma 3.1. Under Assumptions 4 and 5, $ \hat{L}_{n}(\beta)=L_{n}(\beta)+O_{p}(a^{2}_{n}) $.

Theorem 3.2 (Asymptotic Normality). Let $ g(\beta, \gamma)=\sum_{k\,{=}\,1}^{K}p^{*}(k\mid x)v^{\intercal}_{k}(x)\beta_{k} $. Under Assumptions 1-10, for any $ x\in\mathcal{X}\subseteq\mathbb{R}^{p}, $
 

\begin{align*}\sqrt{n}\{\hat{\tau}(x)-\tau^{*}(x)\}\overset{d}{\rightarrow}N(0,(\mathcal{D}g)A^{-1}_{0}B_{0}A^{-1}_{0}(\mathcal{D}g)^{\intercal}),\end{align*}

where $ A_{0}=E[H(\omega, \theta_{0})], $  $ B_{0}=E[\psi(\omega, \theta_{0})\psi^{\intercal}(\omega, \theta_{0})] $, $\mathcal{D}g = [\partial^\intercal g(\beta,\gamma)/\partial \beta $  $\partial^\intercal g(\beta,\gamma)/\partial \gamma]^\intercal$.

Proofs are provided in the Supplementary Material. Lemma 3.1 states that the difference between the oracle (2.2) and plug-in (2.4) loss functions diminishes with rate $ a^{2}_{n} $, which implies that $ \hat{\beta}={\rm arg\min}_{b}n^{-1}\sum_{i\,{=}\,1}^{n}\{Y_{i}-m^{*}(X_{i})-u_{i}^{\intercal}b\}^{2}+O_{p}(a^{2}_{n}). $  Theorem 3.2 provides the asymptotic normality result for the multi-study $ R $-learner estimator $ \hat{\tau}(x) $ for any $ x\in\mathcal{X} $. To examine efficiency gains, we compare the multi-study $ R $-learner to the study-specific $ R $-learner estimator where we fit an $ R $-learner on each study separately and then ensemble the results $ \hat{\tau}^{SS}(x)=\sum_{k\,{=}\,1}^{K}\hat{p}(k\mid x)v_{k}^{\intercal}(x)\hat{\beta}^{R}_{k}, $ where for $ \hat{u}^{R}_{k, i}=\hat{p}(k\mid X_{i})^{-1}\hat{u}_{k, i},\hat{\beta}^{R}_{k}={\rm arg\min}_{b}{n_{k}}^{-1}\sum_{i\,{=}\,1}^{n_{k}}\left[\{Y_{i}-\hat{m}_{k}^{-q_{k}(i)}(X_{i})\}-(\hat{u}^{R}_{k, i})^{\intercal}b\right]^{2}. $ The study-specific $ R $-learner estimator is similar to the multi-study $ R $-learner in that it accounts for between-study heterogeneity in the 1) CATEs, 2) expected potential outcome under no treatment given covariates, and 3) propensity score functions. However, it differs by treating each study separately, without any information shared across studies (ie no cross-study learning).

Theorem 3.3 (Efficiency). Suppose Assumptions 1-10 hold. When $ K\,{=}\,2 $, if for all $ x\in\mathcal{X}, $  $ p^{*}(1\mid x)=p^{*}(2\mid x) $, $ v_{1}(x)=v_{2}(x) $, $ e^{*}_{1}(x)\{1-e^{*}_{1}(x)\}=ce^{*}_{2}(x)\{1-e^{*}_{2}(x)\} $ for $ c\in\mathbb{R}^{+} $ such that $ c\neq 1 $, and $ \sigma^{2} > (1+c)(2\sqrt{c})^{-1} $, then $ var(\hat{\tau}(x))_{\mathrm{Asy}} < var(\hat{\tau}^{SS}(x))_{\mathrm{Asy}}. $

A proof is provided in the Supplementary Material. Theorem 3.3 states that in the two-study setting, the multi-study $ R $-learner is more efficient than the study-specific $ R $-learner when there is between-study heterogeneity in the propensity score functions, and the conditional variance of the error term $ \sigma^{2} $ is greater than the threshold $ (1+c)(2\sqrt{c})^{-1} $. In practice, an example where $ e^{*}_{1}(\cdot)\{1-e^{*}_{1}(\cdot)\}=ce^{*}_{2}(\cdot)\{1-e^{*}_{2}(\cdot)\} $ holds is when the randomization ratios differ between studies. Under Assumption 10, note that $ \sigma^{2}=Var(Y\mid X, A)=\sum_{k\,{=}\,1}^{K}\sigma^{2}_{k}p^{*}(k\mid X)+Var(m_{S} (X)\mid X) $ captures between-study heterogeneity in the mean outcome model $ m_{S}(X) $ across studies $ S\,{=}\,1, \ldots, K. $ As such, $ (1+c)(2\sqrt{c})^{-1} $ is a between-study-heterogeneity transition point beyond which the multi-study $ R $-learner is more efficient.

## 4. OVARIAN CANCER SIMULATIONS

We performed simulations comparing the multi-study $ R $-learner to the study-specific $ R $-learner $ \hat{\tau}^{SS}(\cdot)=\sum_{k\,{=}\,1}^{K}\hat{\tau}^{R}_{k}(\cdot)\hat{p}(k\mid\cdot) $, where $ \hat{\tau}^{R}_{k}(\cdot) $ is the study-specific CATE obtained by fitting an $ R $-learner on study $ k $. To emulate realistic covariate distributions, we randomly sampled $ p\,{=}\,10 $ gene expression covariates from $ K\,{=}\,3 $ studies in the curatedOvarianData R package. For each patient $ i $, we calculated their Mahalanobis distance $ d_{i}=(X_{i}-\hat{\mu})^{\intercal}\hat{\Sigma}(X_{i}-\hat{\mu}), $ where $ \hat{\mu} $ and $ \hat{\Sigma} $ were the sample mean and covariance of $ X $, respectively. Patients with $ d_{i} $ in the bottom 85th percentile were assigned to the training set, and those with $ d_{i} $ in the top 10th percentile to the test set. The middle 5% were discarded to ensure non-overlapping covariate regions between the two sets. We introduced between-study heterogeneity by varying study-specific CATEs $ \tau_{k}^{*}(\cdot) $ across studies, assigned treatment at the individual level, and generated errors $ \epsilon_{i} $ that were independent across patients conditional on $ (X_{i},S_{i},A_{i}) $; thus, the outcomes were conditionally independent within studies. We considered two scenarios for the study-specific CATE functions $ \tau^{*}_{k}(\cdot) $. In Scenario A (linear), $ \tau^{*}_{k}(X_{k})=X_{k}\beta_{k}+Z_{k}\eta_{k} $, where $ X_{k}\in\mathbb{R}^{p} $ denotes study $ k $’s covariates, $ \beta_{k}\sim\mbox{MVN}(0, \mathbb{I}_{p}) $ the fixed effects, and $ Z_{k}\in\mathbb{R}^{q} $ the subset of $ X_{k} $ that corresponded to the random effects $ \eta_{k}\sim\mathrm{MVN}(0, \sigma^{2}_{\tau}\mathbb{I}_{q}) $. Larger $ \sigma^{2}_{\tau} $ corresponded to higher between-study heterogeneity in $ \tau^{*}_{k}(\cdot) $. We generated the true membership probabilities $ p^{*}(k\mid\cdot) $ using a multinomial logistic regression model. In Scenario B (non-linear), we generated highly non-linear study-specific CATEs $ \tau_{k}^{*}(X_{k})=\sin(2X_{k1}+2\eta_{k1})+\log(|X_{k2}+\eta_{k2}|)+\cos(X_{k3}+\eta_{k3})\times(X_{k4}+\eta_{k4})^{2} $ with perturbations $ \eta_{k}\sim\mathrm{MVN}(0, \sigma^{2}_{\tau}\mathbb{I}_{q}) $. As in Scenario A, larger $ \sigma^{2}_{\tau} $ corresponded to higher between-study heterogeneity in $ \tau^{*}_{k}(\cdot). $ To introduce more flexible and non-linear relationships between covariates and study membership, we generated $ p^{*}(k\mid\cdot) $ using a random forest. Across both scenarios, we performed 1000 simulation replicates. We estimated the nuisance functions $ m^{*}_{k}(\cdot),e^{*}_{k}(\cdot), $ and membership probabilities $ p^{*}(k\mid\cdot) $ on the training set using elastic net with cubic-spline expanded covariates and estimated the CATE on the test set. All analyses were performed using R v4.3.1.

**Fig. 1.**
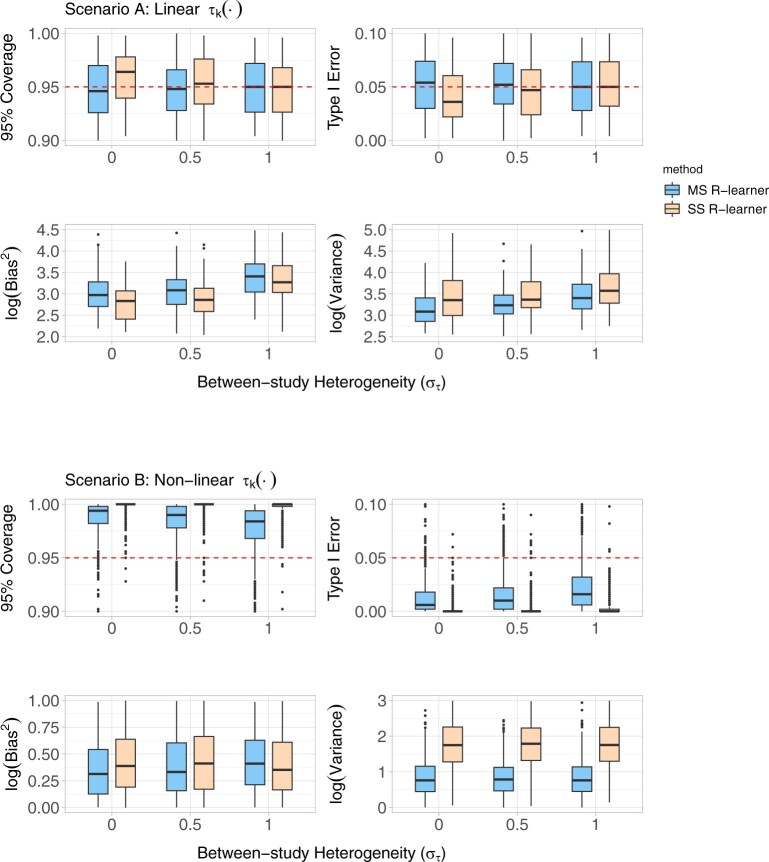
Simulation metrics of the multi-study $ R $-learner (MS $ R $-learner) and study-specific $ R $-learner (SS $ R $-learner) across different levels of between-study heterogeneity in the HTEs ($ \sigma_{\tau} $). Scenario A) Linear $ \tau_{k}(\cdot) $. Scenario B) Non-linear $ \tau_{k}(\cdot). $.

Figure 1 shows the simulation metrics of the multi-study $ R $-learner (blue) and study-specific $ R $-learner (beige) on the test set across different levels of between-study heterogeneity in $ \tau^{*}_{k}(\cdot) $. In Scenario A (linear $ \tau^{*}_{k}(\cdot) $), both approaches maintained nominal 95% coverage and controlled type I error under the null hypothesis that the average treatment effect is zero across all levels of heterogeneity. The multi-study $ R $-learner had slightly higher bias but lower variance compared to the study-specific $ R $-learner. In Scenario B (highly non-linear $ \tau^{*}_{k}(\cdot) $), both approaches tend to be conservative in terms of 95% coverage and type I error control. Bias was comparable across the two approaches. As in Scenario A, the multi-study $ R $-learner generally had lower variance across all levels of $ \sigma_{\tau} $, suggesting that it yielded more precise CATE estimates across both scenarios. Because we did not introduce within-study dependence in our simulations, we used standard variance estimators. If the outcomes were correlated within study or treatment were assigned at the study level as in cluster randomized trials, inference should account for clustering (eg using cluster-robust variance estimators) where there are $ K $ rather than $ n $ independent units.

## 5. BREAST CANCER DATA APPLICATION

We used data from the curatedBreastData R package to illustrate the multi-study $ R $-learner in a realistic setting. Our goal was to estimate the CATE of neoadjuvant chemotherapy for early breast cancer. Clinically, four breast cancer subtypes are defined by estrogen receptor (ER), progesterone receptor (PR), and human epidermal growth factor receptor (HER2) status: Luminal A (ER/PR$ + $, HER2$ - $), Luminal B (ER/PR$ + $, HER2$ + $), HER2$ + $ (ER$ - $, PR$ - $, HER2$ + $), and triple negative (ER$ - $, PR$ - $, HER2$ - $). Because these biologically distinct subtypes respond differently to chemotherapy, estimating HTE is critical for informing treatment strategies. We focused on estimating the effect of anthracycline-based chemotherapy (doxorubicin; $ A\,{=}\,1 $) compared to taxane-based chemotherapy (docetaxel; $ A\,{=}\,0 $), with the outcome defined as pathological complete response ($ Y\,{=}\,1 $ for complete disappearance of cancer, $ Y\,{=}\,0 $ otherwise). For covariates, we used four clinical covariates (age, ER, PR, and HER2 status) that are routinely used in breast cancer treatment decisions and the expression levels of 96 genes implicated in modifying chemotherapy effects (eg *BRCA1/2*) (Sotiriou and Pusztai 2009). We used the transformation $ 2\times\mathrm{expit}(\hat{\tau}(\cdot))-1 $ to map predicted treatment effects onto a [-1, 1] scale, where positive values indicate greater benefit of anthracycline over taxane, and vice versa for negative values.

Our objective was to train the multi-study $ R $-learner and study-specific $ R $-learner using $ K\,{=}\,2 $ studies on early-stage breast cancer (RCT and observational study with $ n\,{=}\,94 $ and 180 patients, respectively) and estimate the CATE on an independent hold-out study (observational study of $ n\,{=}\,261 $ patients). All four breast cancer subtypes (Luminal A, Luminal B, HER2$ + $, and triple negative) were represented in the training data (Table S1 in the Supplementary Materials provides an overview). We estimated the nuisance functions from the training data using elastic net with tuning parameters selected by cross-validation. Because study 1 was an RCT, we used 0.5 as the propensity score. We estimated $ \hat{e}_{2}(\cdot) $ using data from study 2. To estimate the membership probabilities, we fit a logistic regression model to the training data’s study labels.

**Fig. 2.**
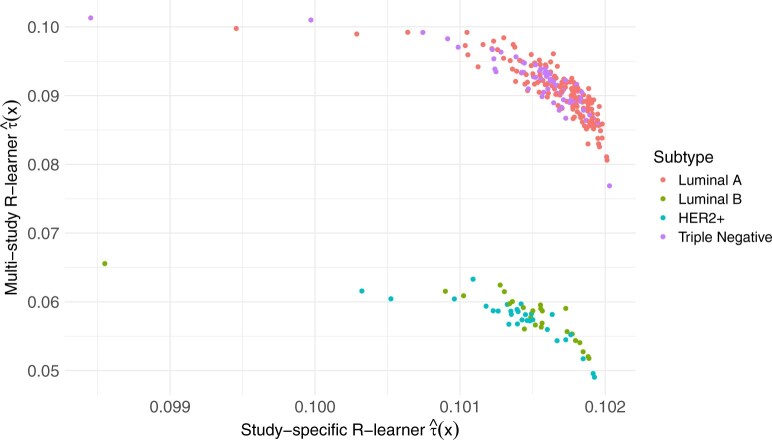
Estimated CATE $ \hat{\tau}(\cdot) $ for breast cancer chemotherapy on the hold-out set. Patients’ CATEs are colored by cancer subtype: Luminal A (ER+ and/or PR+, HER2−), Luminal B (ER+ and/or PR+, HER2+), HER2+ (HER2+, ER−/PR−), Triple Negative (ER−, PR−, HER2−).

Figure 2 shows $ \hat{\tau}(\cdot) $ on the hold-out study stratified by breast cancer subtype. Overall, the study-specific $ R $-learner’s CATEs exhibited minimal variability across subtypes, with most values concentrated between 0.101 and 0.102. This homogeneity likely reflects within-study data sparsity for some subtypes (eg HER2$ + $), which inflates the variance of study-specific $ \hat{\tau}^{R}_{k}(\cdot) $. Cross validation then favors a larger penalty, shrinking $ \hat{\tau}^{R}_{k}(\cdot) $ towards a nearly constant mean effect and masking subtype differences. In contrast, the multi-study $ R $-learner is less sensitive to within-study sparsity because it pools information across studies, resulting in less shrinkage and revealing distinct patterns in the CATEs. Specifically, patients with Luminal A or triple negative tumors had CATEs around 0.08 to 0.10, suggesting greater predicted benefit from anthracycline-based chemotherapy, whereas patients with Luminal B or HER2+ tumors had lower estimated treatment effects around 0.05 to 0.06. These patterns are consistent with prior findings that tumor subtypes differ in their sensitivity to chemotherapy (Rouzier et al. 2005).

## 6. DISCUSSION

The proposed method builds on Nie and Wager (2021)’s $ R $-learner by extending it to multiple studies. To this end, it naturally extends some of the $ R $-learner’s theoretical properties. First, it extends its regret characterization, defined as the excess risk relative to the oracle loss, to the multi-study setting. Specifically, the regret of the multi-study $ R $-learner is $ R(\tau_{1},\ldots, \tau_{K})=L(\tau_{1},\ldots, \tau_{K})-L(\tau^{*}_{1},\ldots, \tau^{*}_{K})=E\left[\sum_{k\,{=}\,1}^{K}e^{*}_{k}(X)\{1-e^{*}_{k}(X)\}p^{*}(k\mid X)^{2}\{\tau_{k}^{*}(X)-\tau_{k}(X)\}^{2}\right] $, which generalizes the overlap-weighted MSE bound introduced in Nie and Wager (2021) to $ K $ studies. Second, the multi-study $ R $-learner admits a flexible reparameterization $ \tau_{k}(\cdot)=\tau(\cdot)+\delta_{k}(\cdot) $, where $ \delta_{k}(\cdot) $ captures study-specific deviations from the overall CATE $ \tau(\cdot) $. Penalizing $ \delta_{k}(\cdot) $ shrinks them toward zero, reducing the multi-study $ R $-learner to the single-study setting with an overall CATE $ \tau(\cdot). $ This reparameterization is useful when between-study heterogeneity in the CATEs is expected to be mild. Conversely, the original parameterization in Equation (2.2) is preferable when between-study heterogeneity is expected to be large. In practice, domain knowledge and computational tools for assessing between-study heterogeneity (eg cross-validated $ R $-loss) can guide the choice between parameterizations.

We offer two considerations on sample size and disentangling effect modifiers for the multi-study $ R $-learner. First, increasing the number of studies (and hence the overall sample size $ n $) generally improves efficiency because the asymptotic variance scales with $ 1/n $. However, the asymptotic variance also depends on the conditional variance $ \mathrm{Var}(Y\mid X, A)=\sum_{k\,{=}\,1}^{K}\sigma_{k}^{2}p(k\mid X)+\mathrm{Var}(m_{S}(X)\mid X), $ where the second term captures between-study heterogeneity in the conditional outcome model $ m_{S}(X)=E[Y\mid X, S] $. If heterogeneity across studies is large, this term can inflate the variance and offset the efficiency gains from the increased sample size. Thus, the ideal number of studies depends on balancing efficiency gains from increased sample size against potential increases in between-study heterogeneity across studies. Second, disentangling effect modifiers from the multi-study $ R $-learner $ \hat{\tau}(\cdot) $ can be approached in several ways. If basis expansions (eg splines) were used in estimation, variable importance measures and partial dependence plots can help disentangle relevant modifiers.

The multi-study $ R $-learner is related to recent generalizations of the $ R $-learner to multi-level and continuous treatments. For multi-level treatments, Acharki et al. (2023) and Zhou et al. (2023) proposed extensions that residualize over treatment-specific indicators. Extending the multi-study $ R $-learner to multi-level treatments involves encoding them as binary vectors and applying a multivariate version of the Robinson-style decomposition as suggested in the Discussion of Nie and Wager (2021). For continuous treatments, Zhang et al. (2022) addressed non-identifiability of the generalized $ R $-loss by introducing a two-step procedure involving $ \ell_{2} $-regularization and a zero-constraining operator. Extending the multi-study $ R $-learner to continuous treatments is a valuable direction that would require adapting this strategy within each study-specific component and weighting them using the estimated membership probabilities.

Another related line of work is combining RCTs with observational studies to estimate causal effects. Approaches such as cross-validated targeted maximum likelihood estimation (CV TMLE) (Dang et al. 2022) and adaptive-TMLE (van der Laan et al. 2024) aim to correct for potential biases when external data are pooled with RCTs. Specifically, CV TMLE uses data-adaptive selection of external controls to mitigate the risk of invalid pooling, and adaptive-TMLE decomposes the target estimand into a pooled treatment effect and a bias term reflecting study enrollment effects. Similarly, Wu and Shu (2021) proposed the integrative $ R $-learner for estimating the CATE in an observational study by leveraging RCT data for identification and de-confounding. Both CV TMLE and integrative $ R $-learner assume mean exchangeability over $ S $, which may not always hold in practice due to between-study heterogeneity in the CATE functions. The multi-study $ R $-learner addresses this by explicitly modeling between-study heterogeneity in the CATEs, and to that end, complements existing approaches by providing a framework for combining RCTs and observational studies while accounting for between-study heterogeneity in the treatment effects. Conversely, these existing approaches offer insightful techniques for bias and confounding correction, which can be incorporated into our framework for future work.

## Supplementary Material

kxaf040_Supplementary_Data

## Data Availability

Data used can be found in R packages curatedOvarianData and curatedBreastData. Code for this paper can be found at https://github.com/cathyshyr/multi-study-r-learner.
