# Supplementary material for: Multi-study R-learner for estimating heterogeneous treatment effects across studies using statistical machine learning
Source: Biostatistics. 2025 Dec 18;26(1):kxaf040. doi: 10.1093/biostatistics/kxaf040 (PMC12713001; doi:10.1093/biostatistics/kxaf040)
Supplement: kxaf040_Supplementary_Data [file kxaf040_supplementary_data.pdf]

*Biostatistics* (2025), **0**, 0, *pp.* **??–??**
doi:10.1093/biostatistics/output

# Supplementary Materials for Multi-Study *R*-Learner for Estimating Heterogeneous Treatment Effects Across Studies Using Statistical Machine Learning

Cathy Shyr[1,*], Boyu Ren[2], Prasad Patil[3], Giovanni Parmigiani[4]

[1]*Department of Biomedical Informatics, Vanderbilt University Medical Center, 2525 West End Ave., Nashville, TN 37203, USA.* [2]*Laboratory for Psychiatric Biostatistics, McLean Hospital, 115 Mills St., Belmont, MA 02478, USA.* [3]*Department of Biostatistics, Boston University School of Public Health, 715 Albany St., Boston, MA 02115, USA.* [4]*Department of Data Science, Dana-Farber Cancer Institute, 450 Brookline Ave., Boston, MA 02215, USA*

*Corresponding author: Cathy Shyr. Email: cathy.shyr@vumc.org

© The Author 2025. Published by Oxford University Press. All rights reserved. For permissions, please e-mail: journals.permissions@oup.com

## 1. Supplementary Material

*Proof.* (Proposition 1)

$$
\begin{aligned}
E[\epsilon \mid X, A] =& E[Y(A) \mid A, X] - E\left[\sum_{k=1}^{K}\left\{\mu_k^{(0)}(X) + A\tau_k(X)\right\} p(k \mid X) \middle| A, X\right] \\
=& E[Y(A) \mid A, X] - \sum_{k=1}^{K} p(k \mid X) E[\underbrace{E[Y(0) \mid X, S=k]}_{\mu_k^{(0)}(X)} \mid A, X] \\
&- A\sum_{k=1}^{K} p(k \mid X) E[\underbrace{E[Y(1) - Y(0) \mid X, S=k]}_{\tau_k(X)} \mid A, X] \\
=& E[Y(A) \mid A, X] - (1-A)\sum_{k=1}^{K} p(k \mid X) E[E[Y(0) \mid X, S=k] \mid A, X] \\
&- A\sum_{k=1}^{K} p(k \mid X) E[E[Y(1) \mid X, S=k] \mid A, X] \\
=& E[Y(A) \mid A, X] - (1-A)\sum_{k=1}^{K} p(k \mid X) E[E[Y(0) \mid A=0, X, S=k] \mid A, X] \\
&- A\sum_{k=1}^{K} p(k \mid X) E[E[Y(1) \mid A=1, X, S=k] \mid A, X] \\
=& 0
\end{aligned}
$$

The second to last equality holds by Assumption 2. The last equality holds because for $A = a \in \{0, 1\}$, we have

$$
\begin{aligned}
& E[Y(a) \mid A=a, X] - \sum_{k=1}^{K} p(k \mid X) E[E[Y(a) \mid A=a, X, S=k] \mid A=a, X] \\
=& E[Y(a) \mid A=a, X] - \sum_{k=1}^{K} p(k \mid X) E[Y(a) \mid A=a, X] \\
=& E[Y(a) \mid A=a, X] - E[Y(a) \mid A=a, X] \\
=& 0.
\end{aligned}
$$

□

*Proof.* (Lemma 3.1) Recall that

$$\hat{L}_n(\beta) = \frac{1}{n}\sum_{i=1}^{n}\left[\{Y_i - \hat{m}^{-q(i)}(X_i)\} - \hat{u}_i^\intercal \beta\right]^2$$

and

$$L_n(\beta) = \frac{1}{n}\sum_{i=1}^{n}\left[\{Y_i - m(X_i)\} - u_i^\intercal \beta\right]^2.$$

We can re-write $\hat{L}_n(\beta)$ as

$$\hat{L}_n(\beta) = \frac{1}{n}\sum_{i=1}^{n}\left[\{Y_i - \hat{m}^{-q(i)}(X_i)\} - \sum_{k=1}^{K}\{A_i - \hat{e}_k^{-q_k(i)}(X_i)\}\hat{p}^{-q(i)}(k \mid X_i) v_k(X_i)^\intercal \beta_k\right]^2,$$

where $\beta_k \in \mathbb{R}^{d_k}$ is the vector of coefficients for study $k$. Similarly, we can re-write $L_n(\beta)$ as

$$L_n(\beta) = \frac{1}{n}\sum_{i=1}^{n}\left[\{Y_i - m(X_i)\} - \sum_{k=1}^{K}\{A_i - e_k(X_i)\}p(k \mid X_i) v_k(X_i)^\intercal \beta_k\right]^2.$$

We define the following notation:

$$\begin{aligned}
A_{m,i} &= m(X_i) - \hat{m}^{-q(i)}(X_i) \\
A_{e_k,i} &= (e_k(X_i) - A_i)p(k \mid X_i) - (\hat{e}_k^{-q_k(i)}(X_i) - A_i)\hat{p}^{-q(i)}(k \mid X_i) \qquad k = 1, \dots, K \\
B_{m,i} &= Y_i - m(X_i) \\
B_{e_k,i} &= (A_i - e_k(X_i))p(k \mid X_i) \qquad k = 1, \dots, K \\
g_k(X_i, \beta_k) &= v_k(X_i)^\intercal \beta_k \\
A_{e,i} &= \sum_{k=1}^{K} A_{e_k,i} g_k(X_i; \beta_k) \\
B_{e,i} &= \sum_{k=1}^{K} B_{e_k,i} g_k(X_i; \beta_k),
\end{aligned}$$

By algebra, we have

$$\begin{aligned}
\hat{L}_n(\beta) =& \frac{1}{n}\sum_{i=1}^{n}\left[B_{m,i} + A_{m,i} - \sum_{k=1}^{K}(B_{e_k,i} + A_{e_k,i})g_k(X_i;\beta_k)\right]^2 \\
=& \frac{1}{n}\sum_{i=1}^{n}\left[B_{m,i} + A_{m,i} - B_{e,i} - A_{e,i}\right]^2 \\
=& L_n(\beta) + \frac{1}{n}\sum_{i=1}^{n}\left[A_{m,i} - A_{e,i}\right]^2 + \frac{2}{n}\sum_{i=1}^{n}(B_{m,i} - B_{e,i})(A_{m,i} - A_{e,i}) \\
=& L_n(\beta) + \frac{1}{n}\sum_{i=1}^{n}A_{m,i}^2 + \frac{1}{n}\sum_{i=1}^{n}A_{e,i}^2 - \frac{2}{n}\sum_{i=1}^{n}A_{m,i}A_{e,i} \\
&+ \frac{2}{n}\sum_{i=1}^{n}B_{m,i}A_{m,i} - \frac{2}{n}\sum_{i=1}^{n}B_{m,i}A_{e,i} - \frac{2}{n}\sum_{i=1}^{n}B_{e,i}A_{m,i} + \frac{2}{n}\sum_{i=1}^{n}B_{e,i}A_{e,i}
\end{aligned}$$

**First term:** $\frac{1}{n}\sum_{i=1}^{n}A_{m,i}^2 = \frac{1}{n}\sum_{i=1}^{n}(m(X_i) - \hat{m}^{-q(i)}(X_i))^2$

By Markov's inequality and Assumption 5, $\frac{1}{n}\sum_{i=1}^{n}A_{m,i}^2$ is $O_p(a_n^2)$.

**Second term:** $\frac{1}{n}\sum_{i=1}^{n}A_{e,i}^2 = \frac{1}{n}\sum_{i=1}^{n}\left[\sum_{k=1}^{K}A_{e_k,i}g_k(X_i;\beta_k)\right]^2$

We have

$$\begin{aligned}
\frac{1}{n}\sum_{i=1}^{n}A_{e,i}^2 = &\sum_{k=1}^{K}\left[\frac{1}{n}\sum_{i=1}^{n}\left\{(e_k(X_i) - A_i)p(k \mid X_i) - (\hat{e}_k^{-q_k(i)}(X_i) - A_i)\hat{p}^{-q(i)}(k \mid X_i)\right\}^2 g_k(X_i;\beta_k)^2\right] \\
&+ \sum_{k\neq k'}\left[\frac{2}{n}\sum_{i=1}^{n}\left\{(e_k(X_i) - A_i)p(k \mid X_i) - (\hat{e}_k^{-q_k(i)}(X_i) - A_i)\hat{p}^{-q(i)}(k \mid X_i)\right\} g_k(X_i;\beta_k)\right. \\
&\left.\times\left\{(e_{k'}(X_i) - A_i)p(k' \mid X_i) - (\hat{e}_{k'}^{-q_k(i)}(X_i) - A_i)\hat{p}^{-q(i)}(k' \mid X_i)\right\} g_{k'}(X_i;\beta_{k'})\right]
\end{aligned}$$

By Markov's inequality and Assumption 5, the first sum is $O_p(a_n^2)$. Similarly, the cross term is also $O_p(a_n^2)$. Therefore, we can conclude that $\frac{1}{n}\sum_{i=1}^{n}A_{e,i}^2$ is $O_p(a_n^2)$.

**Third term:** $\frac{1}{n}\sum_{i=1}^{n}A_{m,i}A_{e,i}$

We have

$$
\begin{aligned}
&\frac{1}{n}\sum_{i=1}^{n} A_{m,i}A_{e,i} \\
&= \frac{1}{n}\sum_{i=1}^{n}\left\{(m(X_i) - \hat{m}^{-q(i)}(X_i))\left[\sum_{k=1}^{K}\left\{(e_k(X_i) - A_i)p(k \mid X_i) - (\hat{e}_k^{-q_k(i)}(X_i) - A_i)\hat{p}^{-q(i)}(k \mid X_i)\right\} g_k(X_i;\beta_k)\right]\right\} \\
&\leqslant \frac{C_1}{n}\sum_{i=1}^{n}\left\{(m(X_i) - \hat{m}^{-q(i)}(X_i))\left[\sum_{k=1}^{K}(e_k(X_i) - A_i)p(k \mid X_i) - (\hat{e}_k^{-q_k(i)}(X_i) - A_i)\hat{p}^{-q(i)}(k \mid X_i)\right]\right\} \\
&\leqslant C_1\sqrt{\left(\frac{1}{n}\sum_{i=1}^{n}\left\{m(X_i) - \hat{m}^{-q(i)}(X_i)\right\}^2\right)\left\{\frac{1}{n}\sum_{i=1}^{n}\left[\sum_{k=1}^{K}(e_k(X_i) - A_i)p(k \mid X_i) - (\hat{e}_k^{-q_k(i)}(X_i) - A_i)\hat{p}^{-q(i)}(k \mid X_i)\right]^2\right\}} \\
&= O_p(a_n^2)
\end{aligned}
$$

for some constant $C_1$. The second line holds by Assumption 4, and the third line holds by Cauchy-Schwarz inequality.

**Fourth term:** $\frac{1}{n}\sum_{i=1}^{n} B_{m,i}A_{m,i} = \frac{1}{n}\sum_{i=1}^{n}(Y_i - m(X_i))(m(X_i) - \hat{m}^{-q(i)}(X_i))$

We define

$$B_{mm}^{q} = \frac{1}{|\{i : q(i) = q\}|}\sum_{i:q(i)=q} B_{m,i}A_{m,i}$$

to be the sample average of $B_{m,i}A_{m,i}$ in the $q$th cross-fitting fold. By the triangle inequality,

$$\left|\frac{1}{n}\sum_{i=1}^{n}(Y_i - m(X_i))(m(X_i) - \hat{m}^{-q(i)}(X_i))\right| \leqslant \sum_{q=1}^{Q}|B_{mm}^{q}|.$$

Therefore, it suffices to show that $B_{mm}^{q} = O_p(a_n^2)$. Let $\mathcal{I}^{-q} = \{X_i, A_i, Y_i, S_i : q(i) \neq q\}$ denote the set of observations that do not belong to the same data fold as observation $i$. $B_{mm}^{q}$'s expectation is

$$
\begin{aligned}
E\left(B_{mm}^{q}\right) &= E(B_{m,i}A_{m,i}) \\
&= E\left(E\left[B_{m,i}A_{m,i} \mid \mathcal{I}^{-q}, X_i\right]\right) \\
&= E(A_{m,i}E\left[B_{m,i} \mid \mathcal{I}^{-q}, X_i\right]) \\
&= 0,
\end{aligned}
$$

where the last line follows from Assumption 1 and Assumption 2.

Next, its variance is

$$
\begin{aligned}
Var\left(B^q_{mm}\right) &= E\left\{\left(B^q_{mm}\right)^2\right\} \\
&= \frac{E\left\{\sum_{i:q(i)=q} B^2_{m,i}A^2_{m,i} + \sum_{i\neq j:q(i)=q,q(j)=q} B_{m,i}B_{m,j}A_{m,i}A_{m,j}\right\}}{|\{i:q(i)=q\}|^2} \\
&= \frac{E\left(B^2_{m,i}A^2_{m,i}\right)}{|\{i:q(i)=q\}|} + \frac{\sum_{i\neq j:q(i)=q,q(j)=q} E\left(B_{m,i}B_{m,j}A_{m,i}A_{m,j}\right)}{|\{i:q(i)=q\}|^2}
\end{aligned}
$$

For the first term, we have

$$
\begin{aligned}
E\left(B^2_{m,i}A^2_{m,i}\right) &= E\left(E\left[B^2_{m,i}A^2_{m,i} \mid \mathcal{I}^{-q}, X_i\right]\right) \\
&= E\left(A^2_{m,i}E\left[B^2_{m,i} \mid \mathcal{I}^{-q}, X_i\right]\right) \\
&\leqslant C_2 E(A^2_{m,i}) \\
&= O(a_n^2)
\end{aligned}
$$

for some constant $C_2$. The second to last line holds from Assumption 4. And the last line holds from Assumption 5.

For the second term, we have

$$
\begin{aligned}
E\left(B_{m,i}B_{m,j}A_{m,i}A_{m,j}\right) &= E\left[E\left(B_{m,i}B_{m,j}A_{m,i}A_{m,j} \mid \mathcal{I}^{-q}, X_i\right)\right] \\
&= E\left[A_{m,i}A_{m,j}E\left(B_{m,i}B_{m,j} \mid \mathcal{I}^{-q}, X_i\right)\right] \\
&= E\left[A_{m,i}A_{m,j}E\left(B_{m,j} \mid \mathcal{I}^{-q}, X_i\right)E\left(B_{m,i} \mid \mathcal{I}^{-q}, X_i\right)\right] \\
&= 0
\end{aligned}
$$

The second to last line follows because $B_{m,i}$ is independent of $B_{m,j}$ for $i \neq j$. The last line follows by the definition of $B_{m,i}$. Therefore, we have that

$$
Var(B^q_{mm}) = \frac{Q}{n}O(a_n^2) = O(a_n^2/n),
$$

where the first equality holds if the $Q$ folds have equal number of observations (i.e., $n/Q$ for each fold). Then by Chebychev's inequality, $\frac{1}{n}\sum_{i=1}^{n} B_{m,i}A_{m,i} = O_p(a_n^2/n)$.

**Fifth and Sixth terms** can be shown to be $O_p(a_n^2/n)$ by a similar argument.

**Seventh term:**

$$\begin{aligned}
\frac{1}{n}\sum_{i=1}^{n} B_{e,i}A_{e,i} &\leqslant \frac{C_4}{n}\sum_{i=1}^{n} A_{e,i} \\
&= \frac{C_4}{\sqrt{n}}\sqrt{\frac{1}{n}\left(\sum_{i=1}^{n} A_{e,i}\right)^2} \\
&= \frac{C_4}{\sqrt{n}}\sqrt{\frac{1}{n}\left(\sum_{i=1}^{n} A_{e,i}^2 + \sum_{i\neq j} A_{e,i}A_{e,j}\right)} \\
&\leqslant \frac{C_5}{\sqrt{n}}\sqrt{\frac{1}{n}\sum_{i=1}^{n} A_{e,i}^2} \\
&\leqslant \frac{C_6}{\sqrt{n}}\sqrt{\left(\frac{1}{n}\sum_{i=1}^{n} A_{e,i}^2\right)\left(\frac{1}{n}\sum_{i=1}^{n} A_{e,i}^2\right)} \\
&= O_p(a_n^2/\sqrt{n})
\end{aligned}$$

for some constants $C_4$, $C_5$, and $C_6$. The first line holds by Assumption 4, and the last line holds by Assumption 5.

Putting all seven terms together, $\hat{L}_n(\beta) - L_n(\beta)$ is dominated by $O_p(a_n^2)$ terms, so $\hat{L}_n(\beta) - L_n(\beta) = O_p(a_n^2)$. □

*Proof.* (Theorem 3.2)

Under Assumptions 1-10, Lemma 3.1, and Lemma 4.1 (Pointwise Linearization) in **?**, we have that

$$\sqrt{n}(\hat{\theta} - \theta_0) = \sqrt{n}\left(\begin{bmatrix}\hat{\beta} - \beta \\ \hat{\gamma} - \gamma\end{bmatrix}\right) = \sqrt{n}\left(\begin{bmatrix}\mathbb{G}_n[u_i\epsilon_i] + o_p(1) + O_p(\sqrt{n}a_n^2) \\ \hat{\gamma} - \gamma\end{bmatrix}\right),$$

where $\mathbb{G}_n[f(w_i)] = \frac{1}{\sqrt{n}}\sum_{i=1}^{n}(f(w_i) - E[f(w_i)])$. Under Assumption 5, $O_p(\sqrt{n}a_n^2)$ is negligible

compared to $o_p(1)$, so we have

$$\sqrt{n}(\hat{\theta}-\theta_0)=\sqrt{n}\left(\begin{bmatrix}\hat{\beta}-\beta\\ \hat{\gamma}-\gamma\end{bmatrix}\right)=\sqrt{n}\left(\begin{bmatrix}\mathbb{G}_n[u_i\epsilon_i]+o_p(1)\\ \hat{\gamma}-\gamma\end{bmatrix}\right).$$

By Theorem 4.2 (Pointwise Normality) in **?**, we conclude that

$$\sqrt{n}(\hat{\theta}-\theta_0)=\sqrt{n}\left(\begin{bmatrix}\hat{\beta}-\beta\\ \hat{\gamma}-\gamma\end{bmatrix}\right)\xrightarrow{d}N(0,A_0^{-1}B_0A_0^{-1})$$

where $B_0=E[\psi(\omega,\theta_0)\psi^{\intercal}(\omega,\theta_0)]$ and $A_0=E[H(\omega,\theta_0)]$. Let $g(\beta,\gamma)=\sum_{k=1}^{K}p(k\mid x)v_k^{\intercal}(x)\beta_k$, we have

$$\sqrt{n}(\hat{\tau}(x)-\tau(x))=\sqrt{n}\left(g(\hat{\beta},\hat{\gamma})-g(\beta,\gamma)\right)\xrightarrow{d}N(0,(\mathcal{D}g)A_0^{-1}B_0A_0^{-1}(\mathcal{D}g)^{\intercal})$$

by the multivariate delta method, where $\mathcal{D}g=\begin{bmatrix}\frac{\partial^{\intercal}g(\beta,\gamma)}{\partial\beta} & \frac{\partial^{\intercal}g(\beta,\gamma)}{\partial\gamma}\end{bmatrix}^{\intercal}$ with derivatives $\frac{\partial g(\beta,\gamma)}{\partial\beta_k}=p(k\mid x)v_k^{\intercal}(x)$ for $k=1,\ldots,K$ and $\frac{\partial g(\beta,\gamma)}{\partial\gamma_k}=v_k^{\intercal}(x)\beta_k x(\zeta_k-p(k\mid x))$ for $k=1,\ldots,K-1$.

□

□

*Proof.* (Theorem 3.3) Suppose the membership probabilities $p(1\mid x)=p(2\mid x)=p$ for all $x$, and $E[\epsilon^2\mid X,A]=\sigma^2$. By Lemma 3.1, Theorem 4.2 (Pointwise Normality) in **?** and Assumptions 8 and 9, we have that for any $\alpha$,

$$\sqrt{n}\alpha^{\intercal}(\hat{\beta}-\beta)\xrightarrow{d}N\left(0,\|s(x)\|_2^2\right),$$

where $s(x)=\Omega^{1/2}\alpha$, $\Omega=\Gamma^{-1}E[\epsilon_i^2u_iu_i^{\intercal}]\Gamma^{-1}$, and $\Gamma=E(u_iu_i^{\intercal})$. Let $\alpha=Z(x)v(x)$, then it follows that

$$\sqrt{n}(\hat{\tau}(x)-\tau(x))\xrightarrow{d}N\left(0,\|s(x)\|_2^2\right).$$

The asymptotic variance of the multi-study $R$-learner estimator $\hat{\tau}(x)$ is

$$\begin{aligned}var(\hat{\tau}(x))_{\text{Asy}}&=\frac{1}{n}v^{\intercal}(x)Z(x)\Omega Z(x)v(x)\\&=\frac{1}{n}v^{\intercal}(x)Z(x)\Gamma^{-1}E[\epsilon^2uu^{\intercal}]\Gamma^{-1}Z(x)v(x)\\&=\frac{\sigma^2}{n}v^{\intercal}(x)Z(x)\Gamma^{-1}Z(x)v(x)\\&=\frac{\sigma^2p^2}{n}v^{\intercal}(x)\Gamma^{-1}v(x)\qquad\text{(S1)}\end{aligned}$$

To obtain the asymptotic variance of the study-specific $R$-learner estimator, i.e.,

$$\hat{\tau}^{SS}(x) = \sum_{k=1}^{K} p v_k^{\intercal}(x) \hat{\beta}_k^R,$$

where $\hat{\beta}_k^R$ is obtained by fitting an $R$-learner to study $k$,

$$\hat{\beta}_k^R = \arg\min_b \frac{1}{n_k} \sum_{i=1}^{n_k} \left[ \{Y_i - \hat{m}_k^{-q_k(i)}(X_i)\} - (u_{k,i}^R)^{\intercal} b \right]^2 + O_p(a_n^2),$$

and $u_{k,i}^R = p^{-1} u_{k,i}$, we use the same strategy as above. Let $\Omega_k^R = (\Gamma_{kk}^R)^{-1} E[\epsilon_{k,i}^2 u_{k,i}^R (u_{k,i}^R)^{\intercal}](\Gamma_{kk}^R)^{-1}$. The asymptotic variance of the study-specific $R$-learner estimator is

$$\begin{aligned}
var(\hat{\tau}^{SS}(x))_{\text{Asy}} &= \sum_{k=1}^{K} \frac{1}{n_k} p^2 v_k^{\intercal}(x) \Omega_k^R v_k(x) \\
&= \sum_{k=1}^{K} \frac{1}{n_k} p^2 v_k^{\intercal}(x) (\Gamma_{kk}^R)^{-1} E[\epsilon_k^2 u_k^R (u_k^R)^{\intercal}] (\Gamma_{kk}^R)^{-1} v_k(x) \\
&= \sum_{k=1}^{K} \frac{\sigma_k^2}{n_k} p^2 v_k^{\intercal}(x) (\Gamma_{kk}^R)^{-1} v_k(x) \\
&= \sum_{k=1}^{K} \frac{\sigma_k^2}{n_k} p^2 v_k^{\intercal}(x) \left( E\left[ \frac{1}{p^2} u_k u_k^{\intercal} \right] \right)^{-1} v_k(x) \\
&= \sum_{k=1}^{K} \frac{\sigma_k^2}{n_k} p^4 v_k^{\intercal}(x) \left( E\left[ u_k u_k^{\intercal} \right] \right)^{-1} v_k(x) \\
&= \frac{p^2}{n} v^{\intercal}(x) \text{blkdiag}\left( \frac{n \sigma_k^2 p^2}{n_k} \Gamma_{kk}^{-1} \right) v(x) \qquad \text{(S2)}
\end{aligned}$$

The goal is to compare (S1) with (S2). We want to show that for all $x \in \mathcal{X}$,

$$\frac{p^2}{n} v^{\intercal}(x) \left( \text{blkdiag}\left( \frac{n \sigma_k^2 p^2}{n_k} \Gamma_{kk}^{-1} \right) - \sigma^2 \Gamma^{-1} \right) v(x) > 0 \qquad \text{(S3)}$$

For $K = 2$, we have

$$\Gamma = E(u_i u_i^{\intercal}) = E \begin{bmatrix} u_{1,i} u_{1,i}^{\intercal} & u_{1,i} u_{2,i}^{\intercal} \\ u_{2,i} u_{1,i}^{\intercal} & u_{2,i} u_{2,i}^{\intercal} \end{bmatrix} = \begin{bmatrix} \Gamma_{11} & \Gamma_{12} \\ \Gamma_{21} & \Gamma_{22} \end{bmatrix}.$$

Let $v_k(\cdot) = v^*(\cdot)$ for all $k$, then

$$\begin{aligned}
\Gamma_{11} &= E[(A_i - e_1(X_i))^2 p^2 v^*(X_i)(v^*(X_i))^\intercal] \\
&= E[e_1(X_i)(1 - e_1(X_i)) p^2 v^*(X_i)(v^*(X_i))^\intercal] \\
\Gamma_{22} &= E[(A_i - e_2(X_i))^2 p^2 v^*(X_i)(v^*(X_i))^\intercal] \\
&= E[e_2(X_i)(1 - e_2(X_i)) p^2 v^*(X_i)(v^*(X_i))^\intercal] \\
\Gamma_{12} = \Gamma_{21} &= E[(A_i - e_1(X_i))(A_i - e_2(X_i)) p^2 v^*(X_i)(v^*(X_i))^\intercal] \\
&= p(\Gamma_{11} + \Gamma_{22})
\end{aligned}$$

Suppose $e_1(\cdot)(1 - e_1(\cdot)) = c e_2(\cdot)(1 - e_2(\cdot))$ for some positive constant $c$, then

$$\Gamma = \begin{bmatrix} \Gamma_{11} & p\Gamma_{11}(1+c) \\ p\Gamma_{11}(1+c) & c\Gamma_{11} \end{bmatrix}.$$

Its inverse is

$$\Gamma^{-1} = \Sigma = \begin{bmatrix} \Sigma_{11} & \Sigma_{12} \\ \Sigma_{21} & \Sigma_{22} \end{bmatrix}$$

where

$$\begin{aligned}
\Sigma_{11} &= (\Gamma_{11} - p^2(1+c)^2 \Gamma_{11}(c\Gamma_{11})^{-1}\Gamma_{11})^{-1} \\
&= \frac{c}{c - p^2(1+c)^2}\Gamma_{11}^{-1} \\
\Sigma_{22} &= (c\Gamma_{11} - p^2(1+c)^2 \Gamma_{11}\Gamma_{11}^{-1}\Gamma_{11})^{-1} \\
&= \frac{1}{c - p^2(1+c)^2}\Gamma_{11}^{-1} \\
\Sigma_{12} = \Sigma_{21} &= \frac{-c}{c - p^2(1+c)^2}\Gamma_{11}^{-1}(p(1+c)\Gamma_{11}(c\Gamma_{11})^{-1}) \\
&= \frac{-p(1+c)}{c - p^2(1+c)^2}\Gamma_{11}^{-1}
\end{aligned}$$

To establish (S3), we want to show that

$$\text{blkdiag}\left(\frac{n\sigma_k^2 p^2}{n_k}\Gamma_{kk}^{-1}\right) - \sigma^2\Gamma^{-1} = \begin{bmatrix} \left(\frac{n\sigma_1^2 p^2}{n_1} - \frac{\sigma^2 c}{c - p^2(1+c)^2}\right)\Gamma_{11}^{-1} & \frac{p(1+c)}{c - p^2(1+c)^2}\Gamma_{11}^{-1} \\ \frac{p(1+c)}{c - p^2(1+c)^2}\Gamma_{11}^{-1} & \left(\frac{n\sigma_2^2 p^2}{n_2 c} - \frac{\sigma^2}{c - p^2(1+c)^2}\right)\Gamma_{11}^{-1} \end{bmatrix}$$

is positive definite. Since $\Gamma$ is positive definite, $\Gamma_{11}^{-1}$ is also positive definite. For $c \neq 1$, $\frac{n\sigma_2^2 p^2}{n_2 c} - \frac{\sigma^2}{c-p^2(1+c)^2}$ is a positive constant, so $\left(\frac{n\sigma_2^2 p^2}{n_2 c} - \frac{\sigma^2}{c-p^2(1+c)^2}\right)\Gamma_{11}^{-1}$ is positive definite. It remains to show that the Schur complement,

$$\left(\frac{n\sigma_1^2 p^2}{n_1} - \frac{\sigma^2 c}{c-p^2(1+c)^2}\right)\Gamma_{11}^{-1} - \left(\frac{p(1+c)}{c-p^2(1+c)^2}\right)^2 \left(\frac{n_2 c(c-p^2(1+c)^2)}{n\sigma_2^2 p^2(c-p^2(1+c)^2) - n_2 c\sigma^2}\right)\Gamma_{11}^{-1}$$

is positive definite. This is equivalent to showing

$$\begin{aligned}
& \frac{n\sigma_1^2 p^2(c-p^2(1+c)^2) - \sigma^2 c n_1}{n_1(c-p^2(1+c)^2)} - \frac{p^2(1+c)^2}{c-p^2(1+c)^2}\left(\frac{n_2 c}{n\sigma_2^2 p^2(c-p^2(1+c)^2) - n_2 c\sigma^2}\right) > 0 \\
\iff \quad & \frac{n\sigma_1^2 p^2(c-p^2(1+c)^2) - \sigma^2 c n_1}{n_1} < \frac{n_2 c p^2(1+c)^2}{n\sigma_2^2 p^2(c-p^2(1+c)^2) - c n_2\sigma^2} \quad \text{(Because } c < p^2(1+c)^2\text{)} \\
\iff \quad & (n\sigma_1^2 p^2(c-p^2(1+c)^2) - \sigma^2 c n_1)(n\sigma_2^2 p^2(c-p^2(1+c)^2) - \sigma^2 c n_2) > n_1 n_2 c p^2(1+c)^2 \\
\iff \quad & n^2\sigma_1^2\sigma_2^2 p^4(c-p^2(1+c)^2)^2 - (n_1\sigma_2^2 + n_2\sigma_1^2)n\sigma^2 p^2 c(c-p^2(1+c)^2) > n_1 n_2 c p^2(1+c)^2 - \sigma^4 c^2 n_1 n_2 \\
\iff \quad & \sigma_1^2\sigma_2^2(np^2(c-p^2(1+c)^2))^2 - (n_1\sigma_2^2 + n_2\sigma_1^2)n\sigma^2 p^2 c(c-p^2(1+c)^2) > -n_1 n_2 c(\sigma^4 c - p^2(1+c)^2)
\end{aligned}$$

Note that the first term is positive and the second is negative. So the left hand side of the inequality is positive. Assuming $\sigma^2 > (1+c)(2\sqrt{c})^{-1}$ for $c \neq 1$, the right hand side is negative.□

| | *Training Data* | | *Evaluation Data* |
|---|---|---|---|
| | **Study 1** | **Study 2** | **Study 3** |
| **GEO Accession** | GSE21997 | GSE25065 | GSE20194 |
| **Study Type** | RCT | Observational | Observational |
| **Sample Size** $N$ | 94 | 180 | 261 |
| **Treatment** $A = 1$ | 54 (57.4%) | 94 (52.2%) | 190 (72.8%) |
| **Outcome** $Y = 1$ | 15 (16.0%) | 42 (23.3%) | 55 (21.1%) |
| **Age** | 52.82 (12.25) | 49.00 (10.29) | 52.20 (10.91) |
| **Tumor Subtype** | | | |
| Luminal A | 44 (46.8%) | 120 (66.7%) | 146 (55.9%) |
| Luminal B | 17 (18.1%) | 1 (0.6%) | 25 (9.6%) |
| HER2+ | 9 (9.6%) | 1 (0.6%) | 29 (11.1%) |
| Triple Negative | 24 (25.5%) | 58 (32.2%) | 61 (23.4%) |

**Supplementary Table S1**. Summary of the studies included in the breast cancer data application. Categorical variables are summarized as $N$ (%); continuous variables are presented as mean (standard deviation). GEO Accession refers to the Gene Expression Omnibus accession number. RCT = randomized controlled trial. Treatment ($A = 1$) indicates treatment assignment to doxorubicin. Outcome ($Y = 1$) corresponds to a pathological complete response, defined as complete disappearance of cancer.

[]
